# Cases of Ischuria

**Published:** 1800-12

**Authors:** 


### *CASES of ISCHURIA.*

### CASE I.

E. S. a corpulent man, aged 67 years, had been ſubject for ſome years to frequent and ſevere attacks of the gout, and nephritic complaints, and had, at different times, voided ſeveral calculi, calculi, was taken, on Tuefday, July the 22d, with fuppreffion of urine.

I was called to him firft on Wednefday the 23d, when he had made only one fpoonful of urine fince yefterday; I ordered him a bolus with opii gr. j. calomel. ppt. camphor aa. gr. v. to be taken immediately, and to be repeated at bed-time; and a mixture with oleum ricini to be taken in the morning.

He had no pain, no inclination to void urine, no fulnefs or tenfion in the bladder, nor any tendernefs upon preffure on the hypogaftrium, but complained of a heavy dull fenfation in his loins; no thirft or fever; pulfe 67, and regular.

Thurfday, 24th. No ftools; no urine. Rept. miftura aper.

Friday, 25th. Has had ftools, but paffed no urine. Applicetur emplaft. veficatorium regioni renum. Rep. bolus h. f.

Saturday, 26th. Capt. cochlear iij. julep; cum fpt. nitros. æther ℥ ia. vel 4 ta. quaq. hora. Rept. bolus h. f. Cras mane rept. miftura aper.

Sunday, 27th. He was electrified by paffing fparks through the region of the kidnies with the large balls of Nairne's patent electrical machine, for fome minutes, and afterwards paffing three fmall fhocks through the loins. Rept. bolus h. f. Cras mane rept. miftura aper.

Monday, 28th. Electricity repeated. He expreffes himfelf to have received a comfortable fenfation from it, as if a weight was removed from his loins. He vomits frequently a green acrid fluid. Rept. bolus h. f.

Tuefday, 29th. The catheter was introduced; no water in the bladder. Electrified again, and vomited immediately after. Bowels regular.

Wednefday, 30th. Two fmall blifters were again applied to the region of the kidnies. Rept. bolus h. f.

Thurfday, 31ft. Would not be electrified any more, as it made him vomit. He is very lethargic; pulfe 57. Rept. bolus h. f.

Friday, Auguft 1ft. Was put into a warm bath for a quarter of an hour. Bowels regular.

Saturday, 2d. Continues the fame, except that his lethargic fymptoms increafe; falls afleep while converfing in an erect pofture. Pulfe low and languid.

Sunday, 3d. About noon he paffed four ounces of urine perfectly clear, after a total fuppreffion for thirteen days, and afterwards paffed a ftone, the fize and form of a fmall kidney bean, without the leaft pain or knowledge of it, till he heard it fall into the chamber-pot.

Monday, 4th. Has voided urine plentifully, of a good colour, without the leaft pain or inconvenience. Has vomited once once this morning, and is rather languid; bowels regular. Rept. bolus f. h. cum calomel. gr. iij.

Tuefday, 5th. Has paffed fome more fmall ftones; vomited this morning.

From this time he continued to void his urine regularly; and on Saturday, the 9th, paffed a ftone larger than the firft. From that time he has advanced in his recovery, and is now, November 4, perfectly well.

### CASE. II.

Friday, Sept. 26, 1800, my fon was called to Tho. Wells, of Fouldon, an old man of 70 years of age, who had had a total fuppreffion of urine from the Sunday preceding.

On examining the abdomen, the bladder was found diftended up to the navel; and, on attempting to pafs the catheter, he could not get it further than the membraneous part of the urethra, where it was plainly felt by the finger introduced into the rectum.

The bladder was pufhed down into the rectum, and behind its own neck, like the retroverted uterus, and was very plainly the obftacle to the introduction of the catheter.

He was bled, and took a bolus with opii gr. j. calomel camphor aa. gr. iij. at five o'clock, and again at bed-time; and had an opening mixture, with oleum ricini, to begin in the morning.

Saturday, the 27th, our friend, Mr. Roberts, of Swaffham, met us, and all our endeavours to pafs the catheter proving fruitlefs, my fon perforated the bladder through the rectum.

As the common trocar would not reach the bladder, he made ufe of Pott's trocar needle and canula, for paffing the feton in the radical operation for the hydrocele.

The catheter being introduced into the urethra as far as it would pafs, he guided the inftrument upon his finger in the rectum, to the protruding bladder, and plunged it into it.

On withdrawing the needle, three pints of urine paffed through the canula, when it ftopped; but trying to pufh forward the catheter, it immediately paffed into the bladder, and three pints more of urine flowed through the catheter.

Sunday, the 28th. He had paffed full two quarts of urine by the urethra, which was tinctured with blood; but none had paffed through the rectum, nor did any afterwards pafs that way.

Monday, the 29th. He had a free paffage through his bowels, and continued for fome days to make a large quantity of urine.

The wound in the rectum and bladder healed by the firft intention, and he gradually recovered, and continues to this time, November 4th, perfectly well.

